# Sonic Hedgehog Activation Is Implicated in Diosgenin-Induced Megakaryocytic Differentiation of Human Erythroleukemia Cells

**DOI:** 10.1371/journal.pone.0095016

**Published:** 2014-04-16

**Authors:** Lamia Ghezali, Bertrand Liagre, Youness Limami, Jean-Louis Beneytout, David Yannick Leger

**Affiliations:** Université de Limoges, FR 3503 GEIST, EA 1069 “Laboratoire de Chimie des Substances Naturelles”, GDR CNRS 3049, Faculté de Pharmacie, Laboratoire de Biochimie et Biologie Moléculaire, Limoges, France; Schulze Center for Novel Therapeutics, Mayo Clinic, United States of America

## Abstract

Differentiation therapy is a means to treat cancer and is induced by different agents with low toxicity and more specificity than traditional ones. Diosgenin, a plant steroid, is able to induce megakaryocytic differentiation or apoptosis in human HEL erythroleukemia cells in a dose-dependent manner. However, the exact mechanism by which diosgenin induces megakaryocytic differentiation has not been elucidated. In this study, we studied the involvement of Sonic Hedgehog in megakaryocytic differentiation induced by diosgenin in HEL cells. First, we showed that different elements of the Hedgehog pathway are expressed in our model by qRT-PCR. Then, we focused our interest on key elements in the Sonic Hedgehog pathway: Smoothened receptor, GLI transcription factor and the ligand Sonic Hedgehog. We showed that Smoothened and Sonic Hedgehog were overexpressed in disogenin-treated cells and that GLI transcription factors were activated. Then, we showed that SMO inhibition using siSMO or the GLI antagonist GANT-61, blocked megakaryocytic differentiation induced by diosgenin in HEL cells. Furthermore, we demonstrated that Sonic Hedgehog pathway inhibition led to inhibition of ERK1/2 activation, a major physiological pathway involved in megakaryocytic differentiation. In conclusion, our study reports, for the first time, a crucial role for the Sonic Hedgehog pathway in diosgenin-induced megakaryocytic differentiation in HEL cells.

## Introduction

Blockage of differentiation or maturation arrest generates myeloid leukemia with genetic lesions in cells. In leukemic cells, this blockage results continuous proliferation, prevention of terminal differentiation and protection from cell death observed in normal blood cells [1]. Differentiation therapy is a potentially less toxic cancer therapy that involves the use of agents, alone or in combination, that modify differentiation and growth of cancer cells [2].

Diosgenin, a plant steroid, has various actions including anti-inflammatory and anti-thrombotic activities as well as anticancer properties [3]–[5]. Previously, *Beneytout et al.* demonstrated that 10µM diosgenin induced megakaryocytic differentiation of HEL cells with increased cell size, nuclear complexity and glycoprotein Ib (GpIb) expression [6]. Recently, we demonstrated that diosgenin-differentiated cells showed nuclear polyploidization and increased expression of the platelet marker CD41 associated with diminution of the erythroid marker glycophorin A (GpA) [7], [8]. Diosgenin induced megakaryocytic differentiation of HEL cells through combined ERK activation and inhibition of the p38 MAPK pathways [7], [9]. Inhibition of ERK activation by a MEK inhibitor abrogated diosgenin-induced differentiation [7].

The Hedgehog (Hh) family of secreted intercellular signaling proteins is essential for the development of many tissues during embryogenesis and is also involved in homeostasis of adult tissues, including skin, gut, bone, and thymus [10]–[14].

In vertebrates, there are three Hh-ligands, Sonic (SHh), Indian (IHh) and Desert Hh (DHh) all closely related to Hh in Drosophila, that regulate a well-defined molecular-genetic signal transduction pathway [15], [16]. The Hh proteins share a common signaling pathway which is initiated when the Hh ligand binds to its cell-surface receptor PATCHED (PTC) which abrogates suppression of the 7-transmembrane-helix protein Smoothened (SMO), the key player for signal transduction of the Hh pathway [17]. SMO activation leads to production of activating forms of the glioma-associated oncoproteins 1–3 (GLI1-3), the Hh transcription factors. Studies on the role of the Hh signaling pathway in hematopoiesis have led to conflicting results. In zebrafish, mutants of the Hh pathway have defects in hematopoietic stem cell (HSC) formation and definitive hematopoiesis [18]. It has also been reported that Hh is implicated in lymphocytic lineage commitment. In fact when PTC1 is suppressed, a defect in the population of the common lymphoid progenitors (CLP) is observed [19].

Given the importance of Hh signaling in tumor biology, a range of drugs has recently emerged to block this signaling pathway. Cyclopamine and jervine, two steroidal alkaloids, extracted from *Veratrum californicum* have a potent inhibitory activity against SMO [20], [21]. Other synthetic compounds have been developed against GLI transcription factors such as GANT-61 which acts downstream of SMO [22]. In a previous study, we showed that cyclopamine, but not jervine, inhibited cell proliferation and induced apoptosis in human erythroleukelia cell lines [23]. Clinical trials investigating the use of Hh inhibitors in patients have recently been initiated for different cancers such as prostate cancer, BCR-ABL–positive acute myeloid leukemia, pancreatic and ovarian cancer [24]–[27]. Because drug resistance appears after long-term Hh inhibition, combining Hh inhibitors with ionizing radiation, chemotherapy or other molecular targeted agents could represent an alternative therapeutic strategy. In fact, different studies suggested that vismodegib (selective hedgehog pathway inhibitor) combined with anticancer drugs increased their respective activities [24], [25].

In this study, we studied the role of SHh during diosgenin-induced megakaryocytic differentiation in the human erythroleukemia cell line HEL. Diosgenin activated SHh production leading to SMO expression and GLI activation. Inhibition of the SHh pathway confirmed that this pathway is involved during diosgenin-induced megakaryocytic differentiation.

## Materials and Methods

### Materials

RPMI 1640 medium, fetal calf serum (FCS) and penicillin/streptomycin were supplied by Gibco BRL (Cergy Pontoise, France). Diosgenin ((25*R*)-5-spirosten-3β-ol), siSMO and primers for RT-PCR were purchased from Sigma Aldrich (Saint Quentin Fallavier, France). GANT-61 was provided by Calbiochem (Fontenay-sous-bois, France).

Phospho-ERK1/ERK2 DuoSet IC ELISA was purchased from R&D Systems (Lille, France). Sonic Hedgehog Human ELISA Kit and SMO antibody were supplied from Abcam (Cambridge, MA). GAPDH antibody was purchased from Santa Cruz Biotechnology (Tebu-Bio, Le Perray en Yvelines, France).

### Cell lines, culture and treatment

The HEL cell line was kindly provided by Professor J.P. Cartron (INSERM U76, Paris, France) and came from ATCC-LGC Standards (Molsheim, France). The TF1a cell line was also purchased from ATCC-LGC Standards. Cells were seeded at 10^5^ cells/ml in tissue culture flasks, grown in RPMI-1640 medium supplemented with 10% FCS, 1% sodium pyruvate, 1% HEPES, 100 U/ml penicillin and 100 µg/ml streptomycin. Cultures were maintained in a humidified 5% CO_2_ atmosphere with at 37 °C. Cells grown for 24 h in culture medium prior to exposure or not to 10 µM diosgenin. For pretreatments 5 µM GANT-61 were added in culture medium for 48 h before subsequent drug treatment. The same amount of vehicle (<0.1% DMSO or ethanol) was added to control cells. Cell viability was determined by trypan blue dye exclusion.

### RNA extraction and semi-quantitative RT-PCR analysis

Total RNA was extracted by RNeasy Mini Kit (Qiagen, Courtaboeuf, France) from treated and control cells. 2 µg of total RNA were transcribed into cDNA using the Omniscript RT kit (Qiagen), and 2 µl of the reverse-transcribed cDNA were used for PCR using the HotStarTaq DNA polymerase mix kit (Qiagen) with 20 pmol of human sense and antisense primers (Table 1).

**Table 1:** Characteristics of RT-PCR primers used for SHh signaling studies.

Target gene	Primer sequence	Size of PCR product	Temperature of hybridation
Human 18S	Forward: GCTGGAATTACCGCGGCTGCT Reverse: CGGCTACCACATCCAAGGAAGG	186	58°C
Human SHh	Forward: CAGTGGACATCACCACGTCT Reverse: CCGAGTTCTCTGCTTTCACC	138	60°C
Human SMO	Forward: CCCATCCCTGACTGTGAGAT Reverse: TTTGGCTCATCGTCACTCTG	176	64°C
Human GLI1	Forward: ACAGCCAGTGTCCTCGACTT Reverse: ATAGGGGCCTGACTGGAGAT	197	60°C
Human GLI2	Forward: GCGTGTTTACCCAATCCTGT Reverse: GATGCTCCCTCAGAGTCCTG	265	60°C

The number of amplification cycles was selected for each gene according to qPCR results, after the Ct value and before plateau levels e.g. during the exponential phase of amplification.

PCR resulting fragments were visualized by electrophoresis on a 1% agarose gel containing ethidium bromide.

### PCR Microarray

Total RNA was extracted by RNeasy Mini Kit (Qiagen, Courtaboeuf, France) from treated and control cells. 1µg of total RNA was transcribed into cDNA using RT^2^ First Strand Kit (QIAGEN) and used for quantitative-PCR according to the RT^2^ profiler PCR array « *human hedgehog signaling pathway* » (QIAGEN). Relative levels of mRNA gene expression were calculated using the 2^−ΔΔCt^ method [28].

### Sonic Hedgehog immunoassay

Sonic Hedgehog production was assessed in cell culture supernatants using the “Sonic Hedgehog Human ELISA kit” (Abcam) according to the manufacturer's instructions.

### Protein expression

After treatment, cells were washed and lysed in RIPA lysis buffer (50 mM HEPES pH 7.5, 150 mM NaCl, 1% deoxycholate, 1% NP-40, 0.1% SDS, 20 µg/ml aprotinin) containing protease inhibitors (Complete Mini, Roche Diagnostics, Meylan, France). Briefly, as previously described [29], proteins (20–50 µg) were separated by electrophoresis on SDS-polyacrylamide gels, transferred to PVDF membranes (Amersham Pharmacia Biotech, Saclay, France) and probed with respective human antibodies against SMO (Abcam, Cambridge, MA), and GAPDH (Santa Cruz Biotechnology, Tebu-Bio, Le Perray en Yvelines, France). After incubation with secondary antibodies (Dako France S.A.S., Trappes, France), blots were developed using the ECL Plus Western Blotting Detection System (Amersham Pharmacia Biotech) and visualized with the G: BOX system (Syngene, Ozyme, Saint-Quentin en Yvelines, France). Membranes were then reblotted with anti-GAPDH used as a loading control.

### Subcellular protein fractionation

After treatment, HEL and TF1a cells were incubated alone or with diosgenin, or GANT-61. Cytosolic and nuclear fractions were obtained using the Subcellular Protein Fractionation Kit according to the manufacturer's protocol (Thermo Fischer Scientific, Rockford, IL, USA) as previously described [30].

### Electromobility shift assay (EMSA)

EMSA experiments were performed using the DIG Gel Shift Kit (Roche Diagnostics). Briefly, nuclear extracts were prepared from cells treated or not with 10µM diosgenin or 5 µM GANT-61. GLI binding reactions were carried out with 5 µg nuclear proteins incubated with digoxigenin (DIG) labeled GLI probe according to the manufacturer's protocol. GLI consensus probe sequence was: forward: 5′-CTCCCGAAGACCACCCACAATGAT-3′, and reverse: 5′-ATCATTGTGGGTGGTCTTCGGGAG-3′. The samples were loaded on a 5% native polyacrylamide gel in Tris-Borate-EDTA buffer. After transfer to nylon membranes and incubation with anti-DIG antibody conjugated with alkaline phosphatase, gel mobility shift was visualized by incubation with CSPD chemiluminescence reagent and detected by the G: BOX system. Quantification of each band was performed by densitometry analysis software with respect to band intensity and band area. Results were expressed relative to controls in arbitrary units.

### Evaluation of nuclear ploidy

For DNA content analysis, after treatment, cells were fixed and permeabilized in 70% ethanol in phosphate-buffered saline (PBS) at −20 °C overnight, washed in PBS, treated with RNase (40 U/μl, Boehringer Mannheim, Meylan, France) for 1 h at room temperature and stained with propidium iodide (PI) (50 µg/ml). Flow cytometry analyses (FC) were performed as previously described [31].

### SMO-specific short-interfering RNA (siRNA) transfection

SMO-specific siRNA (siSMO) were obtained from Sigma Aldrich (Saint-Quentin Fallavier, France) and transfected by electroporation using the AMAXA Nucleofactor system (Lonza, Basel, Switzerland) at 100 nM for 72 h as recommended. SMO silencing was confirmed by western blot.

### Statistical analysis

Data are expressed as the arithmetic means ± standard error of mean (SEM) of separate experiments. The statistical significance of results obtained from *in vitro* studies was evaluated by the two tailed unpaired Student's *t*-test, with *P*<0.05 being considered as significant.

## Results

### Diosgenin modulates Hedgehog gene expression during megakaryocytic differentiation in HEL cells

**Figure 1:**
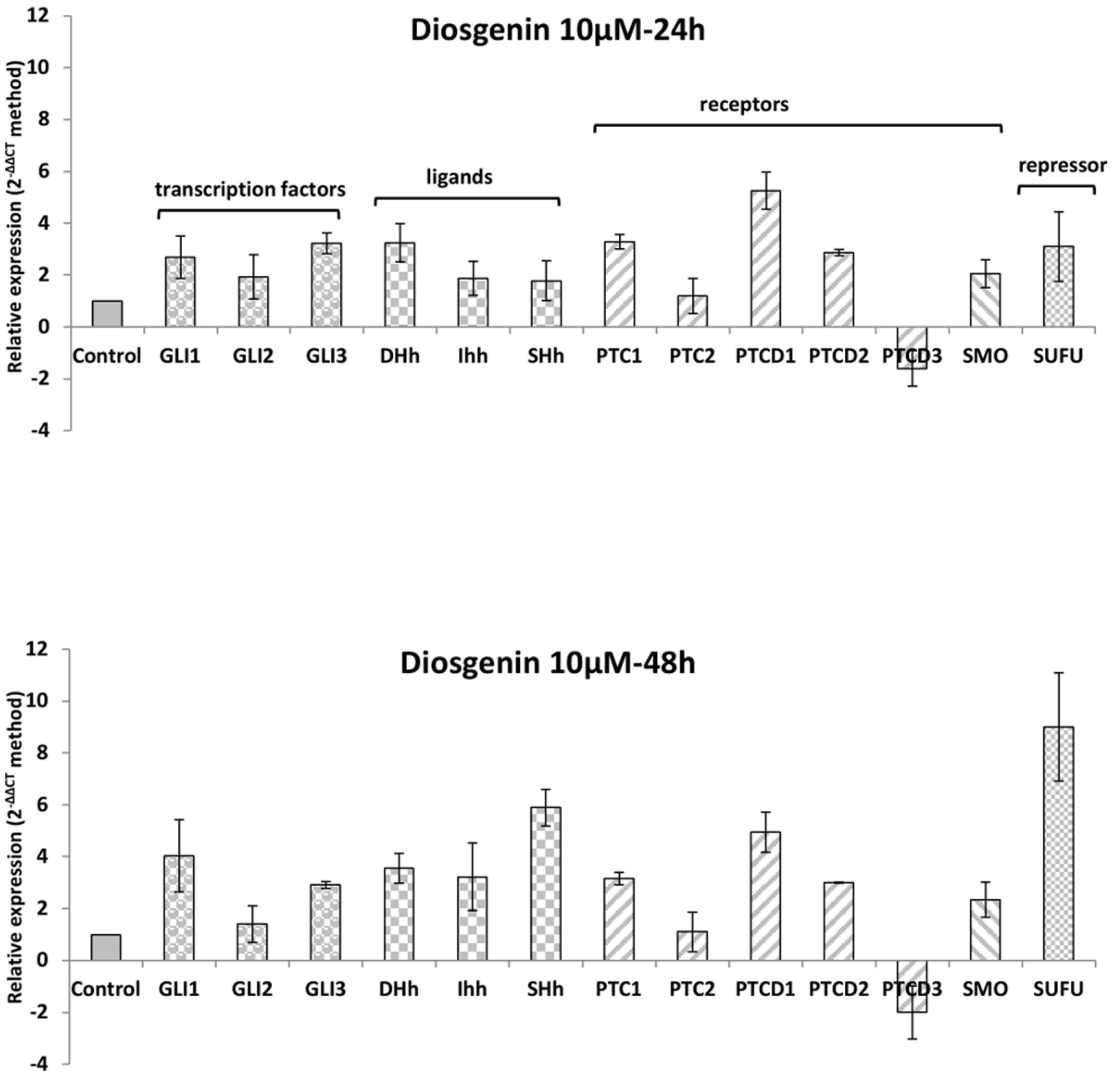
Effect of diosgenin on human Hedgehog signaling pathway gene expression after 24 and 48 h treatment. Cells were treated or not (control) with 10 µM diosgenin for 24 and 48 h then total RNA was extracted. 1µg of total RNA were transcribed into cDNA and used for quantitative-PCR according to the RT^2^ profiler PCR array « human hedgehog signaling pathway ». Relative levels of mRNA gene expression were calculated using the 2^−ΔΔCt^ method versus untreated cells. Each value represents the mean ± SEM of three separate experiments.

First, we analyzed gene expression of major actors of the Hh pathway using the RT^2^ profiler PCR array « *human hedgehog signaling pathway* » (QIAGEN) (Fig. 1). We observed that transcription factors GLI1 and GLI3 were upregulated at 24 and 48 h in diosgenin-treated cells. However, GLI1 upregulation was more pronounced than GLI3 at 48 h, while GLI2 upregulation was not statistically significant. We also found that Hh ligands, DHh, IHh and SHh, were overexpressed at 24 and 48 h. Of note, SHh expression was higher compared to DHh and IHh after 48 h treatment. Receptor genes, including SMO and PTC, were overexpressed except for PTCD3 at both times. As for the negative regulator of the Hh pathway, SUFU, we observed strong upregulation after 48 h treatment.

Taken together, these results suggest that during diosgenin-induced megakaryocytic differentiation in HEL cells, there was an overall upregulation of gene expression of the main actors in the Hh pathway, especially GLI1, SMO, SHh and SUFU.

**Figure 2:**
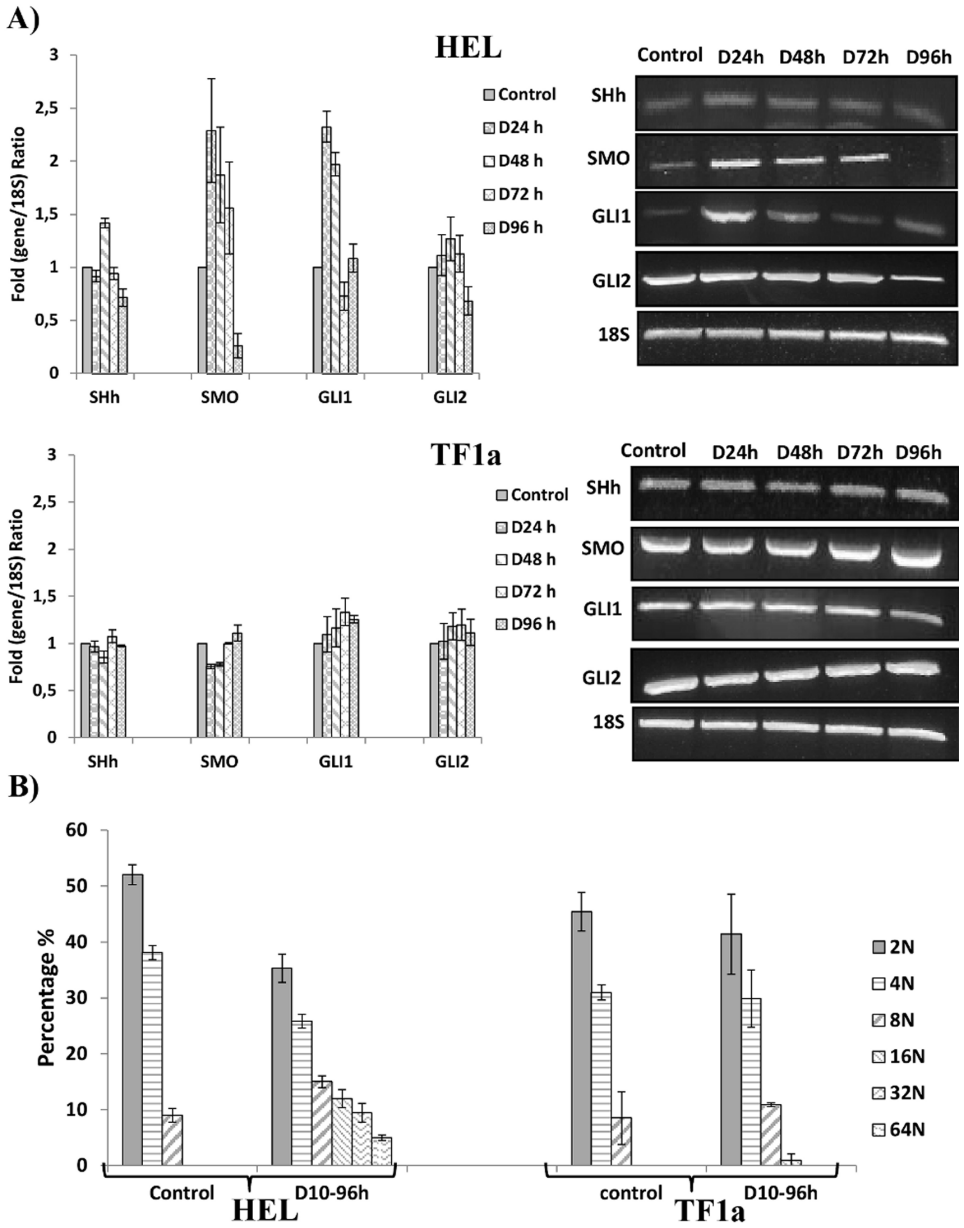
Effect of diosgenin on SHh, SMO and GLI1 gene expression during megakaryocytic differentiation in HEL and TF1a cell lines. (A) Cells were treated or not with 10 µM diosgenin for 24, 48, 72 and 96 h then SHh, SMO and GLI1 genes expressions were evaluated. Total RNA was extracted and 2µg of total RNA were transcribed into cDNA and used for PCR. PCR resulting fragments were visualized by electrophoresis on a 1% agarose gel containing ethidium bromide. Quantification of SHh, SMO and GLI1 transcripts were normalized to 18S as an internal control. The agarose gels shown are representative of six separate experiments. (B) Cells were treated or not with 10µM diosgenin for 96 h and megakaryocytic differentiation was evaluated by analyzing nuclear ploidy. Cells were fixed and permeabilized in 70% ethanol in PBS at −20 °C overnight, washed in PBS, treated with RNase and stained with PI. Then, flow cytometric analyses (FC) were performed to analyze DNA content.

In order to confirm results from the PCR array and to determine whether the SHh pathway was involved throughout diosgenin-induced megakaryocytic differentiation in HEL cells, we analyzed gene expression of SHh, SMO, GLI1 and GLI2 in HEL and in TF1a cell lines after 24 h to 96 h diosgenin treatment (Fig. 2A) by semi-quantitative RT-PCR.

In diosgenin-treated HEL cells, we showed that SHh was overexpressed at 48 h. For other treatment times we did not detect significant changes in gene expression. As for SMO, we found that it was overexpressed from 24 h to 72 h and repressed after 96 h treatment. Furthermore, GLI1 was overexpressed from 24 to 48 h treatment. Of note no significant changes in GLI2 expression were observed.

On the other hand, in diosgnenin-treated TF1a cells, we did not observe any significant changes in SHh, SMO or GLI1-2 gene expression.

Next, we analyzed megakaryocytic differentiation of HEL and TF1a cells after diosgenin treatment by studying nuclear ploidy. Mekagaryocytic differentiation is characterized by progressive polyploidization that ranges from 8N to 128N in normal megakaryocytes. As shown in Fig. 2B, diosgenin increased nuclear ploidy up to 64N in HEL cells after 96 h. In contrast, diosgenin did not induce increased nuclear ploidy in TF1a cells.

Our data document that only HEL cells were able to differentiate after diosgenin treatment with activation of the SHh pathway. In TF1a cells, diosgenin did not induce mergakaryocytic differentiation or SHh pathway activation. We speculated then, that the SHh pathway was involved in diosgenin-induced megakaryocytic differentiation.

### Diosgenin induces SHh-N production, SMO overexpression and GLI activation

**Figure 3:**
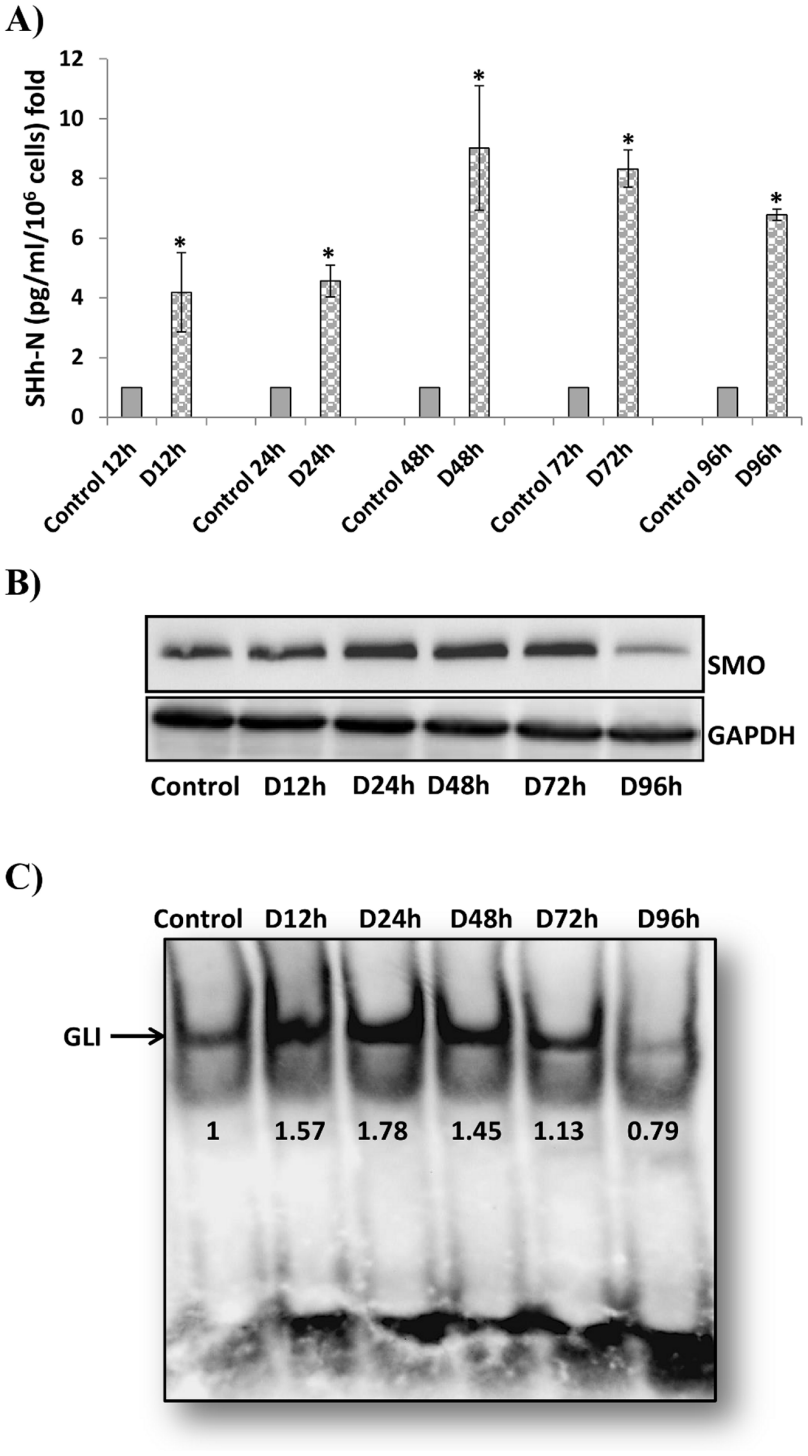
Effect of diosgenin on SHh-N production, SMO expression and GLI1 activation in HEL cells. Cells were treated with 10 µM diosgenin for 12, 24, 48, 72 and 96 h. (**A**) SHh-N production was evaluated in cell culture supernatanst using the “Sonic Hedgehog Human ELISA Kit”. Each value represents the mean ± SD of three separate experiments, * P< 0.05 diosgenin *vs.* control. (**B**) SMO expression was evaluated by Western blot analysis after diosgenin treatment. GAPDH was used as a loading control. (**C**) GLI1 activation was evaluated by electromobility shift assay using the DIG Gel Shift Kit. The blots shown are representative of five separate experiments.

SHh protein is synthesized as a precursor of about 45 kDa, followed by autoproteolytic cleavage which generates an amino-terminal peptide that is linked to cholesterol at its carboxyl terminus. All signaling activities are mediated by this N-terminal peptide, but the C-terminal region is necessary to catalyse the autoproteolytic cleavage event [32]. We checked the effect of diosgenin on SHh-N production using the ‘Sonic Hedgehog Human ELISA Kit’. As shown in Fig. 3A, 10 µM diosgenin induced strong and sustained SHh-N production and secretion, starting at 12 h. This result suggests that the SHh pathway is activated during diosgenin-induced megakaryocytic differentiation in HEL cells.

Because secreted SHh-N can have autocrine or paracrine activity in physiological conditions we studied the activation of the SHh signaling pathway at different stages in diosgenin-treated cells. It is well known that in the presence of Hh ligands, the inhibitory activity of PTC on the positive transmembrane effector SMO is lost and ultimately results in modulation of the activity of the three GLI zinc finger transcription factors at target promoters [33]. SMO overexpression was confirmed at the protein level by western blotting as shown in Fig. 3B. This overexpression lasted from 12 h to 72 h after diosgenin treatment. Next, we examined the effect of diosgenin on GLI1 nuclear activation by gel shift (Fig. 3C). Our results demonstrated that 10 µM diosgenin increased GLI transcriptional activity starting at 12 h as shown by increased DNA binding to a consensus probe. This activation was prolonged until 48 h and then decreased at the end of HEL differentiation.

### SHh pathway is implicated in megakaryocytic differentiation induced by diosgenin

**Figure 4:**
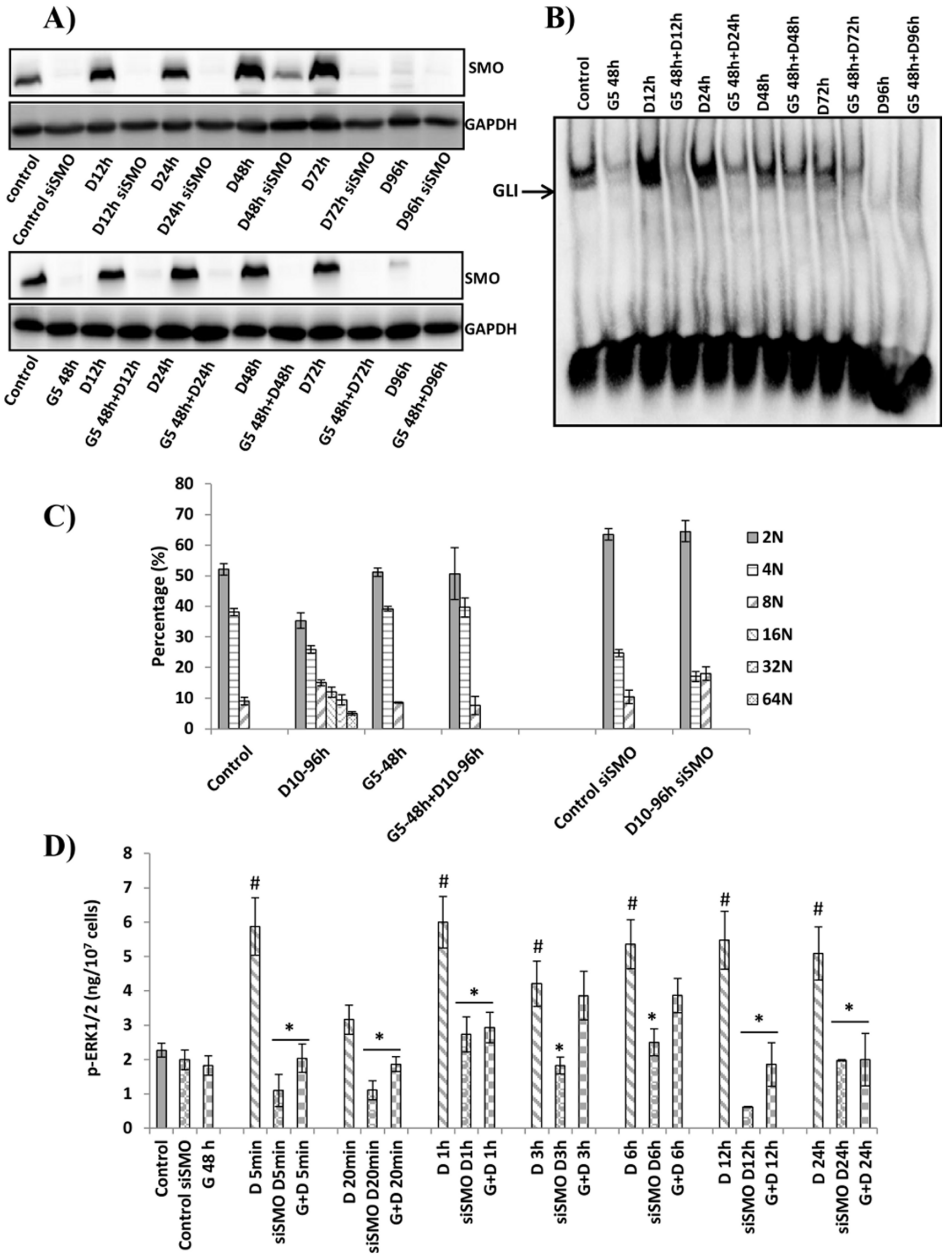
Effects of SMO and GLI1 inhibition on diosgenin-induced megakaryocytic differentiation in HEL cells. (**A**) Cells were transfected with siSMO or pretreated with 5µM GANT-61 for 48 h (G5) then treated with 10 µM diosgenin for 12, 24, 48, 72 and 96 h.SMO expression was evaluated by Western blot. GAPDH was used as a loading control. (**B**) Cells were pretreated with 5µM GANT-61 (G5) then treated with 10 µM diosgenin for 12, 24, 48, 72 and 96 h. GLI1 activation was evaluated by electromobility shift assay using the DIG Gel Shift Kit. The blots shown are representative of five separate experiments. (**C**) Cells were transfected with siSMO or pretreated with 5µM GANT-61 (G5) then treated with 10 µM diosgenin for 96 h. Megakaryocytic differentiation was assessed by analyzing nuclear ploidy. (**D**) Cells were transfected with siSMO or pretreated with 5µM GANT-61 (G5) then treated with 10 µM diosgenin for 5, 20 min, 1, 3, 6, 12 and 24 h. ERK1/2 phosphorylation was quantified using DuoSet IC assay kit. Each value represents the mean ± SEM of three separate experiments, * P< 0.05 siSMO+diosgenin or GANT-61+ diosgenin *vs*. diosgenin, # P< 0.05 diosgenin *vs*. control.

As we showed that the SHh pathway was activated by diosgenin, we wanted to determine whether diosgenin-induced megakaryocytic differentiation of HEL cells was SHh-dependent. We used SMO siRNA or 5 µM GANT-61 during 48 h as a pretreatment. GANT-61 is a small molecule that specifically inhibits GLI [34]. Inhibition of SMO expression after silencing with siRNA or GANT-61 pretreatment was confirmed by western blotting (Fig. 4A) and inhibition of GLI1 activation after GANT-61 pretreatment was assessed by gel shift (Fig. 4B). After confirmation of SMO silencing and GLI inhibition we showed that, in these conditions, diosgenin failed to increase nuclear ploidy to more than 8N, and that cell ploidy remained principally 2N and 4N whereas diosgenin alone increased nuclear ploidy up to 64N (Fig.4C). As polyploidization is a hallmark of megakaryocytic differentiation, this illustrated that sustained SMO and GLI activation was involved in diosgenin-induced megakaryocytic differentiation.

### SMO silencing or GANT-61 pretreatment inhibit ERK1/2 activation

A recent study provided evidence for GLI3 and GLI1 as novel substrates of MAP-kinases (MAPKs) including the MEK substrate ERK2 [35]. On the other hand, it has been shown that Hh-binding to PTC1 stimulates ERK1/2 activation [36] Furthermore, it is also known that ERK1/2 activation is essential in normal mekacaryocytopoeisis and during its induction by diosgenin on HEL cells [7]. As shown in Fig. 4D, SMO silencing or GANT-61 pretreatment inhibited ERK1/2 activation induced by diosgenin. This result indicated that in our model SHh pathway activation acted upstream upstream to ERK.

## Discussion

Differentiation therapy has emerged as a powerful method to target specific hematologic malignancies. One of the best examples is the use of retinoic acid which was one of the first substances used [37]. The mode of action of differentiating agents, whether chemical or natural, is generally to enable or activate signal transduction pathways normally activated by the binding of hematopoietic factors and promote transcription of genes regulating hematopoiesis [38]. Diosgenin is a natural product able to induce megakaryocytic differentiation in the erythroleukemia cell line HEL [4]–[6], but its mode of action is still unclear.

In the present report, we focused our interest on Hh pathways. These signaling pathways represent strong candidate targets to eradicate leukemia, as they convergently regulate hematopoietic stem cell proliferation and have been identified as putative modulators of leukemia stem cell behavior [39], [40]. Of these pathways, SHh signaling is gaining considerable attention as a therapeutic target for myeloid malignancies [40], [41], despite the fact that its role in normal and malignant human hematopoiesis remains poorly defined and controversial [42]–[44].

In our study, we showed that diosgenin induced SHh-N production in HEL cells which activated the SHh pathway. Indeed, this was confirmed by SMO overxpression and GLI activation. It is well known that in the presence of Hh ligands, the transmembrane receptor PTC releases its inhibition of another transmembrane protein SMO, allowing SMO to assume an active conformation. In this activated state, SMO transduces signals that cause the nuclear translocation of the GLI family of transcription factors, with ultimate influences on cell cycling [45].

The SHh protein undergoes an autocatalytic processing reaction that involves internal cleavage, the amino-terminal product of this cleavage receives a covalent cholesterol adduct and becomes active in signaling [46]. The diosgenin structure is related to that of cholesterol. It can be suggested that diosgenin could mimic the effect of physiological cholesterol and bind to SHh-N which in turn would active signal transduction. However, to date no direct binding of diosgenin to SHh-N has been demonstrated, further studies should address this point.

Megakaryocytopoiesis is a highly regulated phenomenon that involves a wide spectrum of cytokines and growth factors in physiological conditions. The role of Hh proteins in the regulation of hematopoiesis has proven controversial, with different experimental models supporting opposing interpretations. A role for IHh in erythropoiesis has been proposed based on *ex vivo* assays: the addition of recombinant IHh to visceral endoderm-depleted epiblasts reinstated vasculogenesis and primitive erythropoiesis, suggesting that IHh may be responsible for haematopoietic stem cell activation and differentiation during primitive haematopoiesis [47]. In adults, SHh has already been implicated in diverse types of differentiation such as osteogenesis [48]–[50].

There is a little evidence to demonstrate the importance of the SHh pathway in differentiation therapy for the treatment of hematological neoplasms. Here we characterized the blockage of megacaryocyctic differentiation induced by diosgenin in HEL cells after blocking the SHh pathway by siSMO. An antagonist of GLI (GANT-61) was also used to further examine SHh implication in diosgenin-induced megakaryocytic differentiation. Inhibition of two different targets of the SHh pathway blocked megakaryocytic differentiation induced by diosgenin in HEL cells.

In different studies, treatment with cyclopamine, a specific SMO inhibitor, induces monocytic differentiation [51] or eosinophilic differentiation [52] of HL-60 cells, but inhibition of the SHh pathway by cyclopamine was also implicated in blockage of erythroid differentiation [53].

As previously described, diosgenin induced megakaryocytic differentiation of the HEL cell line through sustained ERK activation and inhibition of p38 MAPK pathways [7]. In this study, we showed that SHh inhibition blocked diosgenin induced megakaryocytic differentiation and down-regulated ERK1/2 phosphorylation. It is well known that SHh activation leads to MAPK MEK-1/ERK pathway activation [54]. In addition, MEK-1 activation was shown to synergize with the canonical Hh pathway resulting in significant enhancement of GLI-dependent transcriptional activation [54].

Here, SHh pathway inhibition appears to affect megakaryocytic differentiation induced by diosgenin in HEL cells. Further investigations should determine the clinical applications of modulating the SHh pathway in treatment of hematological malignancies.

## Conclusion

**Figure 5:**
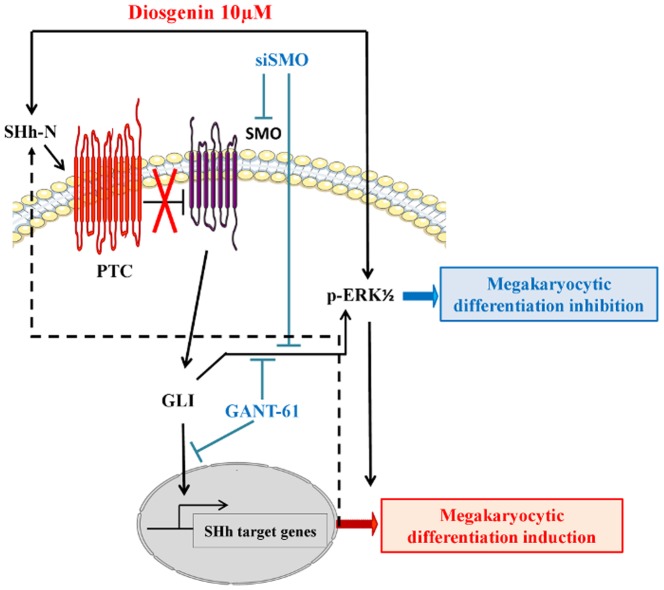
Megakaryocytic differentiation mechanisms in diosgenin-treated cells in relation to the SHh pathway. Diosgenin induced megakaryocytic differentiation in HEL cells. The SHh pathway is involved in diosgenin-induced megakaryocytic differentiation since its inhibition blocks this phenomenon. SMO inhibition by total silencing using siRNA or GLI1 inhibition using GANT-61, a specific inhibitor, blocked megakaryocytic differentiation induced by diosgenin. In addition, SHh pathway inhibition leads to ERK1/2 inhibition whose activation is absolutely required to induce megakaryocytic differentiation.

Our data document, for the first time, a crucial role for the Sonic Hedgehog pathway in diosgenin-induced megakaryocytic differentiation in HEL cells. Inhibiting key actors of this pathway, SMO and GLI, blocked diosgenin-induced megakaryocytic differentiation (Fig. 5). Furthermore, SHh pathway inhibition led to ERK1/2 inhibition (Fig. 5) whose activation was absolutely required to induce megakaryocytic differentiation as previously described [9].
